# Optimization of the size and location of the FOVs for CBCT capture of the TMJ

**DOI:** 10.1186/s12903-025-06081-4

**Published:** 2025-05-10

**Authors:** Marc Anton Fuessinger, Maximilian Frederik Russe, Leonard Simon Brandenburg, Marc Christian Metzger, Johannes Schulze, Stefan Schlager, Wiebke Semper-Hogg

**Affiliations:** 1 Department of Oral and Maxillofacial Surgery, Albert-Ludwigs University Freiburg, Hugstetterstr. 55, 79106 Freiburg, Germany; 2 Department of Oral and Maxillofacial Surgery, Albert-Einstein University Ulm, Albert-Einstein-Allee 11, 89081 Ulm, Germany; 3 Department of Radiology, Albert-Ludwigs University Freiburg, Hugstetterstr. 55, 79106 Freiburg, Germany

**Keywords:** Cone beam CT (CBCT), Field of view (FoV), Imaging

## Abstract

**Background:**

Osseous pathologies of the temporomandibular joint (TMJ) such as degenerative joint disease, trauma, and deformity contribute to orofacial morbidity and are considered a major factor in temporomandibular dysfunction. Cone beam computed tomography (CBCT) is a recommended diagnostic tool in imaging of osseous tissue pathologies. However, CBCT contributes to patient radiation exposure, and limiting the CBCT field of view (FOV) may reduce it. This study aims to investigate the possibility and clinical applicability of optimizing the size and location of the FOVs for CBCT capture of the TMJ.

**Methods:**

Three-dimensional CBCT data sets in which the bilateral positions and dimensions of the TMJs were analyzed. A total of 201 data sets with 402 condyles were mapped in relation to the CBCT device. By transformation into a common coordinate space using the device’s chin rest as a joint denominator, we were able to determine the optimal size and location for uni- and bilateral capture of the TMJ for both best-case and worst-case scenarios with regard to patient positioning.

**Results:**

The minimal FOVs for unilateral capture were H 28.2 mm × R 22.9 mm in the best-case scenario assuming optimal patient positioning and H 47.0 mm × R 28.3 mm in the worst-case scenario with rotational deviation along the transversal axis. For bilateral capture, we determined the best-case FOV as H 24.9 mm × R 66.5 mm and the worst-case FOV as H 42.8 mm × R 66.7 mm.

**Discussion:**

This research yields indication-specific FOVs for both uni- and bilateral imaging of the TMJ. Considering the best clinical practices for CBCT imaging, clinically feasible FOV dimensions in consideration of the technical specifications of common CBCT devices can be suggested. The clinical application of the results may help reducing radiation exposure of patients receiving CBCT imaging of the TMJ. The transferability of the present results to other CBCT devices requires further research.

**Trial registration:**

The study is registered in the German Trial Register with the number DRKS00026149, 2024/02/21.

## Introduction

The temporomandibular joint (TMJ) is a uniquely complex joint [1]. Osseous pathologies of the TMJ are associated with limitations on joint movements, joint pain, and temporomandibular dysfunction [2, 3]. The most common pathologies of the TMJ in adults are degenerative joint diseases (DJD), mainly osteoarthritis and osteoarthrosis [4]. Typical radiologic changes in the morphology of the TMJ have been shown to significantly correlate with pain intensity [5]. Other common indications for the imaging of the TMJ include intra-articular fractures, ankylosis, and hyper- and hypoplasia of the condyles [6].

Compared to panoramic imaging and magnetic resonance imaging (MRI), cone beam computed tomography (CBCT) facilitates the detection of osseous tissue changes in the TMJ [7, 8]. Its diagnostic accuracy is comparable to conventional computed tomography (CT), and it is a suggested mode of extended imaging for many of these pathologies [6, 9–11].

Depending on the specific CBCT device, the size of the field of view (FOV) in which image data is acquired, and the region of interest (ROI), patients may be exposed to lower amounts of ionizing radiation compared to CT imaging [12–21]. CBCT systems vary in the size of the FOV they can capture [22]. Most systems allow the operator to choose from a range of FOVs and possible ROIs. However, there is currently no published data on ROI-specific, patient-independent FOVs for the dedicated imaging of the TMJ.

The aim of the retrospective study was to determine whether it is possible to define a common optimal FOV for CBCT imaging of the TMJ, using a large set of pre-existing CBCT images.

## Methods

### Patient selection

CBCT images of the lower jaw were collected from patients who had undergone scanning for diagnostic purposes. The CBCT scans were mainly obtained for implant surgery, surgical removal of impacted teeth, or orthodontic treatment. Thus, the subjects in the study were not exposed to unnecessary radiation. All images were captured using the 3D Accuitomo F170 (Morita, Japan) [23].

Patients were included if their CBCT scans showed the entire lower jaw including both TMJs. Patients were excluded from the study if they presented any pathology or deformity in the imaged skeleton or surrounding soft tissue that could potentially affect their positioning in the CBCT device. Additionally, exclusion criteria encompassed instances of injury or illness in the TMJ, such as:

1. Congenital deformities: Patients with abnormally shaped or positioned TMJs due to birth defects were excluded.
2. Osteoarthritis: Patients with degeneration of the cartilage and bones in the TMJ, causing changes in the joint’s shape and structure, were excluded.
3. Traumatic injuries: Patients with fractures or dislocations of the TMJ due to facial trauma, resulting in structural damage and deformities, were excluded.
4. Ankylosis: Patients with fused TMJ bones, severely limiting or completely preventing jaw movement, were excluded.
5. Tumors: Patients with tumors in or around the TMJ, causing deformities and affecting joint function, were excluded.
6. Developmental disorders: Patients with conditions such as Pierre Robin sequence or hemifacial microsomia, involving abnormalities in the development of the jaw and TMJ, were excluded.
7. Idiopathic condylar resorption: Patients with gradual breakdown and resorption of the condyle, leading to changes in the joint’s structure and bite alignment, were excluded.

**Fig. 1:**
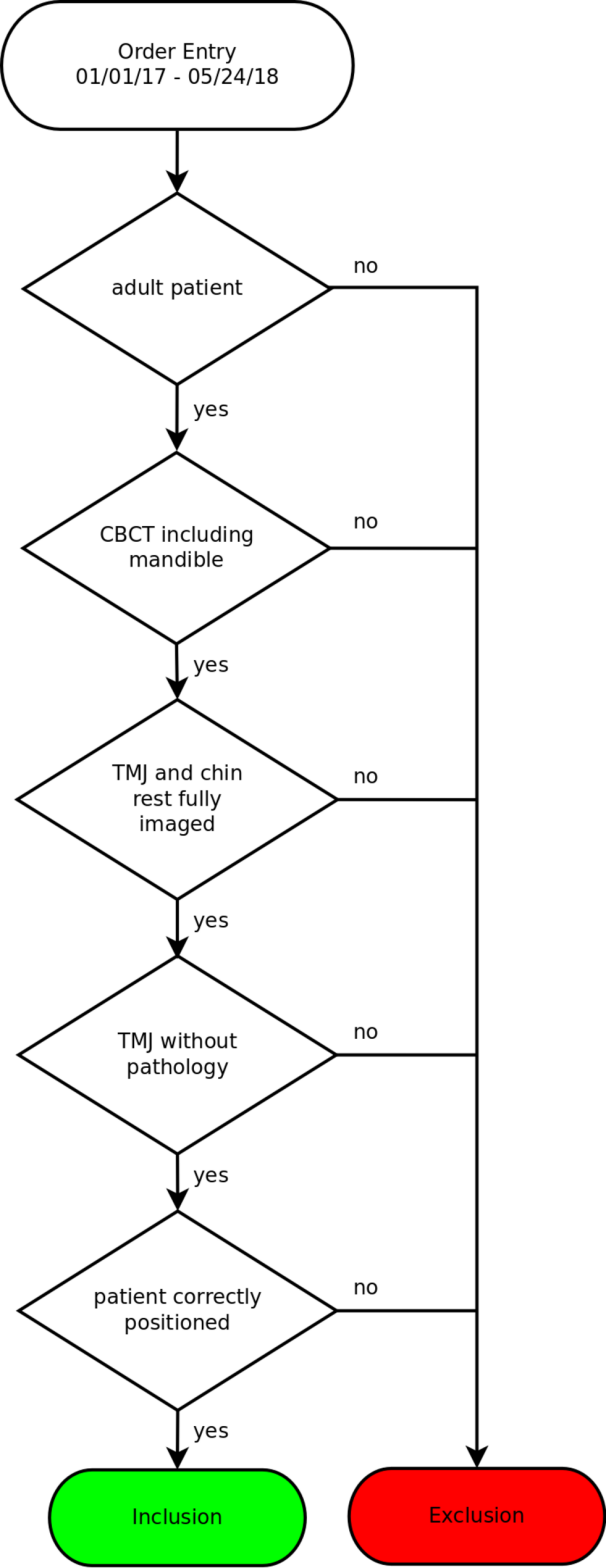
Selection process

The selection process is illustrated in Fig. 1.

From the hospital image data, 201 patients met the criteria. The baseline characteristics of the study population are outlined in Table 1.

**Table 1:** Study population

		Total	Male	Female
**All**		201	87	114
**Age**	18–30 years	139	61	26
	> 30 years	62	78	36
	Mean	45.0	44.5	45.5
	SD	19.4	18.4	20.3
	p-value		0.75	

### Data acquisition

The CBCT images of included patients were anonymized and exported into the DICOM format before conversion into the NIFTI format. NIFTI has a simpler file structure compared to DICOM. A NIFTI file consists of a header and a data array, making it easier to read, write, and manipulate the image data using research software tools and programming languages. The transformation from DICOM to NIFTI format should not inherently impact data resolution or image fidelity.

**Fig. 2:**
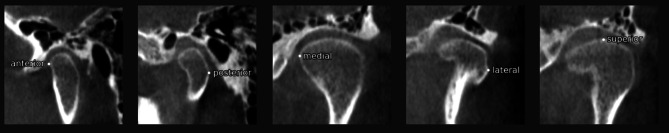
Mapping of the outer boundaries of the condyle in sagittal (**a, b**) and coronal (**c, d, e**) reconstruction

For every pair of TMJs in these images, the dimensions and locations of the joints by placing anatomical marker points in the most anterior, posterior, medial, lateral, and cranial extremities of the mandibular condyles were determined, as depicted in Fig. 2, using the software 3D-Slicer [24]. Two observers independently placed the landmarks on the images to ensure reliability and consistency in the measurements. However, it is important to note that no statistical analysis was performed to assess the inter-observer variability or agreement in the placement of these landmarks. The chin rest of the CBCT device was additionally mapped as a common reference for the location of the anatomical landmarks relative to the CBCT device. The data was augmented with the contra-lateral counterparts mirrored along the midsagittal plane of the data set’s coordinate system to control for asymmetries.

### Data alignment

A single dataset was randomly chosen and manually aligned optimally along the midsagittal plane, the Frankfurt plane, and the base of the mandible. This dataset was used as a reference that all following transformations were based on.

For data alignment, we applied two strategies representing both a worst- and a best-case-scenario for the positioning of the patient in the CBCT device:

Translation only: Using the chin rest corners as a common reference, all datasets were translated into a common coordinate system. As there is no rotational correction around the chin rest corners, this scenario contains all the variability due to suboptimal patient positioning (*worst case*).

Translation and rotation: To control for the variability of the head inclination in the CBCT device due to suboptimal patient positioning, the data sets were additionally rotated around a transversal axis defined by the mapped corners of the chin rest, by minimizing the least square distance between landmark coordinates (*best case*).

### Acquisition of FOVs

The aligned and overlayed data sets resulted in a point cloud of anatomical landmarks. Longitudinally oriented cylinders were placed and optimized to contain the landmark points for the entirety of the data sets as well as differentiated by different groups. The longitudinal orientation of the cylinders is selected to align with the general orientation of the TMJs and to facilitate the subsequent optimization process. The optimization of cylinder dimensions is a crucial step in the process, involving an iterative algorithm that adjusts the cylinder dimensions, including diameter and height, to minimize the distance between the landmark points and the cylinder surface. The objective of this optimization process is to determine the smallest possible cylinder dimensions that still encompass all the landmark points, ensuring that the resulting field of view (FOV) will capture the entire TMJ region for the majority of patients (98th percentile). Furthermore, the optimization process involves differentiating the cylinder dimensions based on the different sex groups.

The cylinder position was mapped as a vector from the midpoint between the chin rest corners of the reference data set to the center of the cylinder base.

### Statistical analysis

Due to the inapplicability of parametric testing procedures, statistical differences between sex and age groups for height, radius, and FOV positions were assessed using a permutation test procedure with 999 random distributions of group assignments.

*Study registration: *The study was authorized by the ethics committee of the Albert Ludwig University Freiburg with the identification number 21-1188 and was registered in the German Clinical Study Registry (DRKS) under the identification number DRKS00026149.

## Results

**Fig. 3:**
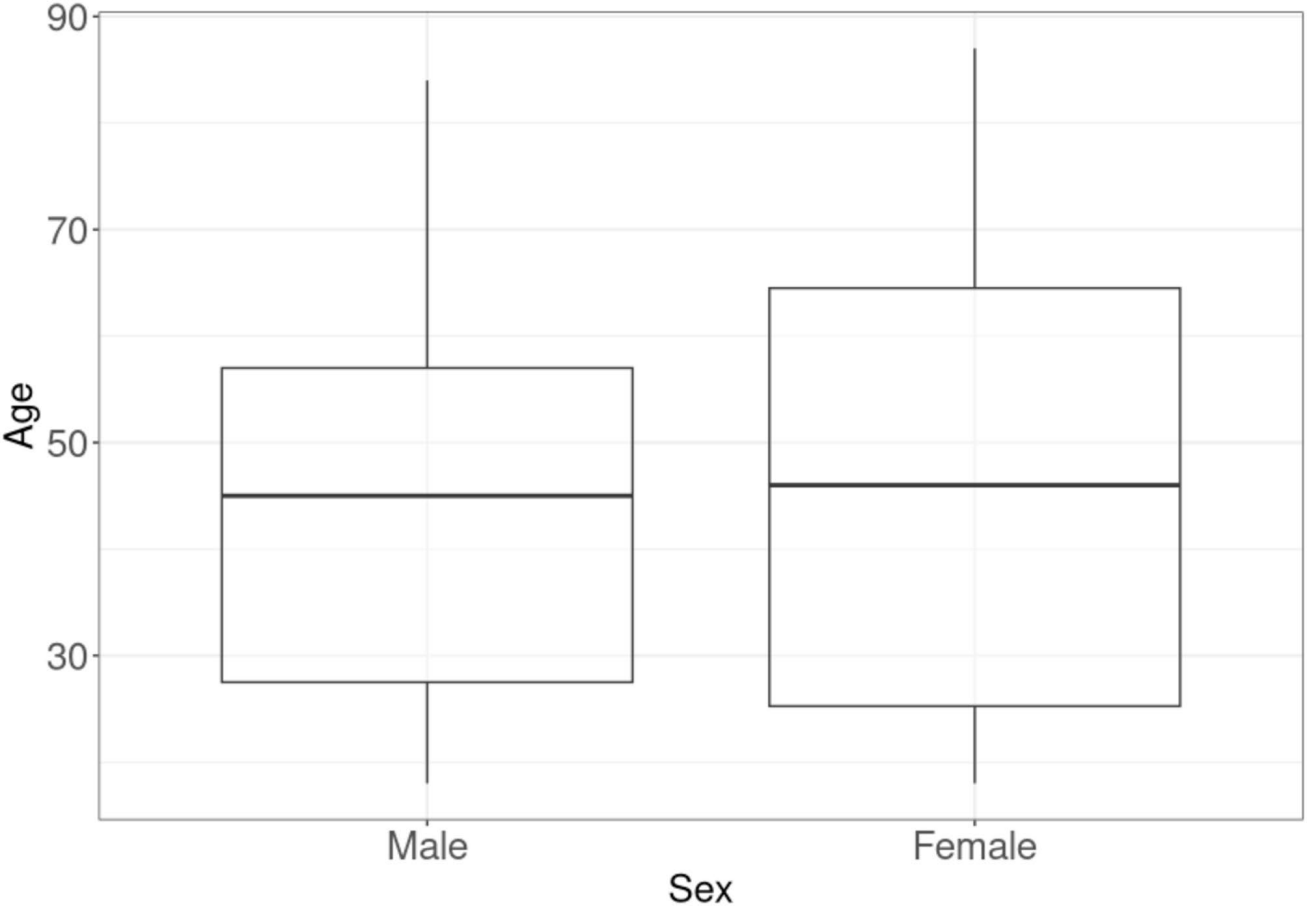
Age distribution of the study population

There were no statistically significant differences in group composition between the male and female subgroups with regard to patient age. The mean age is 45 (± 19.4) years. The age distribution is depicted in Fig. 3.

**Fig. 4:**
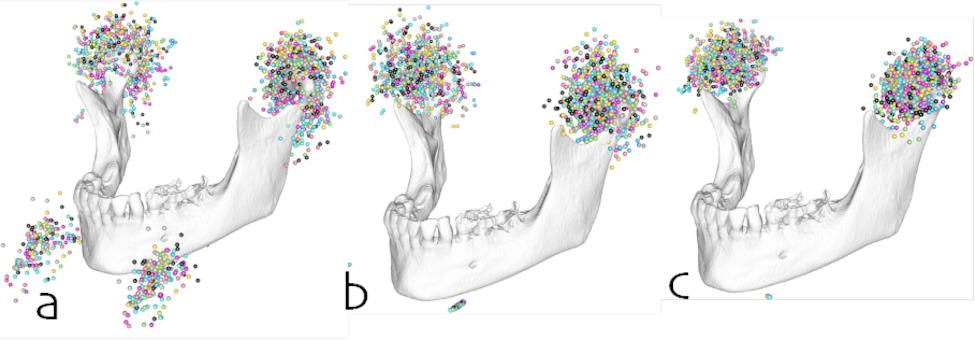
Markups for the TMJ and the chin rest (**a**) before spatial alignment, (**b**) after spatial alignment by translation to a common coordinate system, and (**c**) after spatial alignment by translation to a common coordinate system and rotation around a transversal axis defined by the chin rest

The point clouds of the raw data, the worst-case, and the best-case alignment are depicted in Fig. 4.

The dimensions and locations of the unilateral FOVs do not differ significantly between sexes.

The sex-independent FOVs for the unilateral capture were identified to have the dimensions (height × radius) H 28.2 mm × R 22.9 mm (best case) and H 47.0 mm × R 28.3 mm (worst case).

The dimensions and locations of the bilateral FOVs do not differ significantly between sexes either.

The sex-independent FOVs for the bilateral capture were identified to have the dimensions (height × radius) H 24.9 mm × R 66.5 mm (best case) and H 42.8 mm × R 66.7 mm (worst case).

**Fig. 5:**
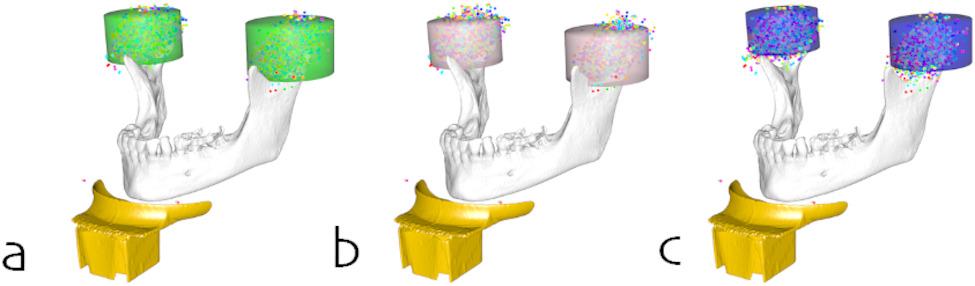
FOVs for unilateral imaging of the TMJ for (**a**) the total study population, (**b**) female, and (**c**) male subjects

**Fig. 6:**
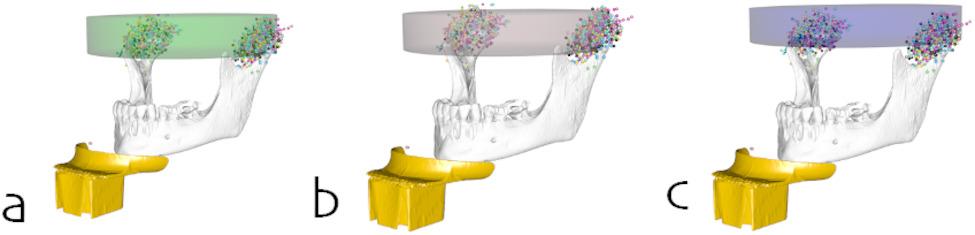
FOVs for bilateral imaging of the TMJ for (**a**) the total study population, (**b**) female, and (**c**) male subjects

Figures 5 and 6 show the resulting FOV cylinders for the total population as raw data and differentiated by sex for the uni- and bilateral capture, respectively. Table 2 contains the dimensions (height and radius) of the cylinders differentiated by the best- and worst-case scenarios while Table 3 contains the positions of the cylinders relative to the chin rest.

**Table 2:** Dimensions of the FOV cylinders for the best- and worst-case scenarios

		Best case		Worst case	
		Radius [mm]	Height [mm]	Radius [mm]	Height [mm]
**All**	Unilateral	22.9	28.2	28.3	47.0
	Bilateral	66.5	24.9	66.7	42.8
**Female**	Unilateral	22.1	26.2	27.5	41.5
	Bilateral	64.4	22.2	64.7	36.2
**Male**	Unilateral	22.0	24.2	27.1	44.7
	Bilateral	68.5	21.4	68.9	40.9

**Table 3:** Position of the FOV cylinder relative to the center of the chin rest along the transversal (X), sagittal (Y), and longitudinal (Z) axis [mm]

		X	Y	Z
**All**	Unilateral	49.97	–80.18	54.90
	Bilateral	0.00	–80.18	54.88
**Female**	Unilateral	48.92	–76.80	53.23
	Bilateral	0.00	–76.80	53.22
**Male**	Unilateral	51.37	–84.55	60.35
	Bilateral	0.00	–84.55	60.25

Since bilateral data sets are generated by mirroring along the midsagittal plane of the coordinate system, the transversal coordinate for bilateral FOVs is always 0.

There is, however, a statistically significant difference between the age groups for the unilateral and bilateral FOVs regarding the dimensions and the cylinder positions in the best-case scenario (Table 4). The FOVs for study participants < 30 years of age were significantly larger in size, in both radius and height for the unilateral FOVs (H 27.6 mm × R 22.5 mm vs. H 23.1 mm × R 20.5 mm) and only in height (H 27.4 mm vs. H 23.1 mm) for the bilateral FOVs. The difference in location of the unilateral FOVs did not cause a significant difference in the radius of the bilateral FOVs.

**Table 4:** Age-specific dimensions and location difference for unilateral FOVs in the best-case scenario

	Age [years]		*p*-value
	< 30	> 30	
Radius [mm]	22.5	20.5	0.002*
Height [mm]	27.6	23.1	0.009*
Location [mm]	4.9		0.02*

*Statistically significant

## Discussion

CT imaging of the TMJ is an important diagnostic approach in the management of temporomandibular disorders and other osseous abnormalities of the TMJ [6, 25, 26]. The *Diagnostic Criteria for Temporomandibular Disorders* recommend TMJ imaging for the confirmation of clinical diagnoses in selected cases and require CT imaging for a diagnosis of DJD [25]. CBCT has been shown to be equivalent to CT in both sensitivity and specificity for the detection of osseous lesions in the mandibular condyle, and the European guidelines on radiation protection recommend CBCT instead of CT as the radiation dose of CBCT is shown to be lower [27, 28]. The therapeutic implications following a CBCT scan remain uncertain [6].

The widespread adoption of CBCT leads to an increased radiation exposure [29]. The effective dose (ED) can be significantly reduced by changes in exposure time, tube current, tube voltage, and voxel size [17, 30]. Usage of low-dose CBCT protocols does not seem to have a negative impact on the diagnostic performance [31]. Optimization of the FOV is one of the main determining factors in reducing radiation exposure [17, 18, 27]. The FOV-dependent ED is affected by both the size (height and diameter) of the FOV and its location. The FOV position relative to the radiation of sensitive organs such as the lens of the eye, the thyroid, and the salivatory glands significantly contributes to the ED [32–34]. Owing to the mainly vertical layout of these organs in the head-and-neck region, the FOV height is also a nonlinear factor in overall radiation exposure [18].

As of 2020 there were at least 279 different CBCT device models available, providing a variety of FOVs [22]. But there are currently no publications examining ROI-specific, patient-independent FOVs for CBCT imaging of the TMJ. There is also no published evidence on the usage of anatomical landmarks for determining FOV positions in TMJ imaging.

Multiple studies have examined the effect of the FOV size on the radiation exposure in CBCT imaging of the TMJ [32, 33, 35–37]. Lukat et al. [32] demonstrated a nearly tenfold reduction in ED when using two separate small FOVs for bilateral TMJ imaging, although their design was limited by the use of different CBCT devices for small and large FOVs. Nascimento et al. [33] and Pinto et al. [37] were also able to demonstrate a significant reduction in exposure when using two small FOVs for bilateral imaging, compared to a single large FOV or bilateral medium-sized FOVs, emphasizing the importance of the FOV choice.

Additionally, the diagnostic sensitivity for pre-existing and artificial osseous lesions of the condyle has been shown to increase with smaller FOVs [35, 38]. This has to be contrasted by the findings by Hung et al. [39] who demonstrated an increased risk of incomplete FOV coverage during CBCT imaging of the TMJ, resulting in a higher frequency of retakes, although their case count was very low.

Using Accuitomo presets with a 4 × 4 volume for TMJ imaging is suboptimal, as the volume size and position do not adequately capture the TMJ; while a larger volume would better focus on the TMJ, it would also increase the patient’s radiation exposure.

The utilization of scout views of anatomical landmarks for individualized positioning aids in adjusting the FOV to suit the patient’s specific anatomy. This requires additional radiation exposure and increases the operational complexity. We decided to calculate the position and dimension of the FOV without the use of anatomical landmarks, to ensure patient-independent FOVs. FOV positions were calculated relative to the chin rest of the devices.

Limitations resulting by patient movement or variability in head posture during the CBCT scanning process must be mentioned. A rigid fixation of patients head together with a precise positioning of the patient n the CBCT device is crucial to translate the results in clinics. To address this issue, we implemented a data processing step that involved rotating the CBCT data sets around a transversal axis. This axis was defined by the mapped corners of the chin rest, which served as a fixed reference point across all patients. The rotation was performed to minimize the least square distance between landmark coordinates, effectively aligning the data sets to a standardized orientation.

This reference allowed us to superimpose all condylar markups into a common coordinate system. The FOVs calculated from these markups correspond to a worst-case scenario, comprising likely imperfect patient positioning mainly in the craniocaudal direction. Rotation of the markups around a transversal axis defined by the chin rest allowed us to determine FOVs for a hypothetical best-case scenario with perfect patient positioning. However, this best-case scenario carries the risks of misalignment of FOV positions and underestimation of the FOV dimensions, especially along the vertical axis. In some patients, this might in practice result in an incomplete FOV coverage and the necessity of a retake. Anatomical features, like special vertical skeletal growing patterns or inflammatory related response of the joint tissues may lead to a non-representation of relevant temporomandibular joint structures. (Lo Giudice 2020, lo Giudice 2018, Lo Giudice 2020)

All imaging used in this study was carried out using the Accuitomo 170 by J. Morita Corp [23]. It appears to be a reasonable and pragmatic approach to translate the results to other manufacturers’ devices using phantom heads and different chin rests, but this has yet to be thoroughly demonstrated. Different CBCT machines may have variations in their hardware, software, and image acquisition protocols, which can lead to differences in the achieved optimization of the field of views (FOVs). These variations can potentially impact image quality, resolution, and the extent of anatomical coverage. As a result, the specific findings and conclusions drawn from this study, which relied on the Accuitomo 170, may not be immediately generalizable to other CBCT devices. Additionally, the CBCT images used in this study were taken as in clinical routine in maximal occlusion, edge-to-edge bite, or with an intermaxillary spacer. Analyzing CBCTs only in maximal occlusion might be part of future investigations. The present study design using a predefined chin rest did not allow for the inclusion of open-mouth imaging of the TMJ. Our results are therefore probably not yet applicable to this type of diagnostics.

To reduce the impact of outliers, the analyses were limited to the 98th percentile of all data points, but entire data sets were not excluded. This may have caused a distortion of the results with an overrepresentation of anomalous condyles. Without the current distortion of results, the FOV could still be reduced.

The approach to determine the extent of the TMJ was limited to the osseous boundaries of the mandibular condyles, not including the joint space and the osseous articular surface on the temporal bone. Due to the cylindrical shape of the FOV there is a risk of incomplete imaging of atypically shaped condyles (Le Giudice). The joint space between the condylar surface and the glenoid fossa has been measured using CBCT in subjects with both normal TMJ function and craniomandibular dysfunction. Al-Rawi et al. [40] measured a mean superior joint space of 6.0 mm, a mean medial and lateral joint space of up to 5.5 mm, and a mean posterior joint space of up to 6.4 mm in edge-to-edge bite. Dalili et al. [41] and Ikeda et al. [42, 43] measured much smaller joint spaces but did not specify the type of intercuspidation. But as CBCT is not suitable for proper soft-tissue diagnostics, it might be reasonable to restrict the FOV to the depiction of bony structures.

Following guidelines for dental imaging, an additional 2-mm margin should be considered to ensure sufficient capture of neighboring structures [44]. To ensure complete imaging of the joint space and the adjoining articular surface would thus require a significant enlargement of the FOV.

Using the determined optimal FOVs and in consideration of the above-mentioned necessary augmentations of the FOV, clinically applicable FOVs with regard to common device specifications can be proposed. Adding a horizontal margin of at least 6.5 mm to the radius and a vertical margin of at least 6 mm to the height of the FOV is suggested. The resulting proposed FOVs are depicted in Fig. 6. The recommended FOVs for unilateral imaging are between commonly used small- and medium-sized FOVs in both the worst- and best-case scenarios. In contrast, the suggested FOVs for bilateral imaging would be categorized as large FOVs. Any imaging using these dimensions should be performed with attention to optimal patient positioning and in maximal occlusion, to reduce the risk of incomplete FOV coverage. In consideration of the dosimetry data by Nascimento et al. [33], additional research is required to determine whether to use one single FOV or two separate FOVs for bilateral imaging of the TMJ.

An influence of the patients’ sex on either the dimensions or locations of indication-specific FOVs could not be documented. However, an influence of the patients’ age was seen, with patients being less than 30 years of age requiring larger and more cranially positioned FOVs. This appears to be in line with research showing continued remodeling of both the ramus and the condyle during aging [42, 45].

Clinical implications: According to the current German guideline, CBCT-based TMJ diagnostics are limited to degenerative inflammatory arthropathies, malpositioned TMJ heads, and fractures [46]. The reliable achievement of precise, preferably small FOVs is an important step in this regard. Enabling dental practitioners to perform TMJ imaging with a radiation dose similar to that of a panoramic radiograph significantly enhances patient care. Presets with the specified FOV could facilitate the workflow and guarantee proper imaging by a reduced radiation dosage [32, 47]. Future research must be done to proof the concept with different CBCT scanners. As a result of the present findings most of the patients TMJs (98th percentile) could be evaluated using the smallest FoV resulting a reduced radiation dosage. However, for the remaining 2% of cases where the TMJs are not completely covered by the smallest FOV, a repeated scan using a scout view may be necessary to ensure that the entire TMJ region is adequately visualized.

Limitations: The applicability of the present findings is further restricted by the study population and the CBCT scanner. There is no testing on phantom models using different CBCT scanners. The study design did not allow for the collection of biometric (e.g. body height, weight) or ethnic data on the included patients. It is reasonable to assume that our study population data set is representative of the general population of South-Western Germany. Evidence on the impact of ethnicity on TMJ anatomy is weak and inconclusive [48–51].

## Conclusion

The practical implementation of the present findings in commercial CBCT systems holds the potential to significantly diminish radiation exposure, thereby enhancing patient care. Further exploration should aim to assess adaptability across various commercially accessible CBCT devices and diverse demographic populations in a mutli-center study.

## Data Availability

The data presented in this study are available on request from the corresponding author. The data are not publicly available due to ethical permission.

## Abbreviations

CBCT: Cone-beam computed tomography
CT: Computed tomography
DJD: Degenerative joint disease
ED: Effective dose
FOV: Field of view
MRI: Magnetic resonance imaging
ROI: Region of interest
TMJ: Temporomandibular joint
